# Impairments of GABAergic transmission in hippocampus mediate increased susceptibility of epilepsy in the early stage of Alzheimer’s disease

**DOI:** 10.1186/s12964-024-01528-7

**Published:** 2024-02-22

**Authors:** Rui Mao, Mengsha Hu, Xuan Liu, Lei Ye, Bingsong Xu, Min Sun, Siyi Xu, Wenxuan Shao, Yi Tan, Yun Xu, Feng Bai, Shu Shu

**Affiliations:** 1 Department of Neurology, Nanjing Drum Tower Hospital, Affiliated Hospital of Medical School, Nanjing University, Nanjing, China; 2 Department of Neurology, Nanjing Drum Tower Hospital Clinical College of Nanjing University of Chinese Medicine, Nanjing, China; 3 Department of Neurology, Nanjing Drum Tower Hospital, State Key Laboratory of Pharmaceutical Biotechnology and Institute of Translational Medicine for Brain Critical Diseases, Nanjing University, Nanjing, China; 4 Jiangsu Key Laboratory for Molecular Medicine, Medical School of Nanjing University, Nanjing, China; 5 Jiangsu Provincial Key Discipline of Neurology, Nanjing, China; 6 Nanjing Neurology Medical Center, Nanjing, China

**Keywords:** Alzheimer's disease, Epilepsy, Cognition, GABAergic transmission, Parvalbumin interneurons, NRG1, ErbB4

## Abstract

**Background:**

Patients with Alzheimer’s disease (AD) are often co-morbid with unprovoked seizures, making clinical diagnosis and management difficult. Although it has an important role in both AD and epilepsy, abnormal γ-aminobutyric acid (GABA)ergic transmission is recognized only as a compensative change for glutamatergic damage. Neuregulin 1 (NRG1)-ErbB4 signaling can promote GABA release and suppress epileptogenesis, but its effects on cognition in AD are still controversial.

**Methods:**

Four-month-old APPswe/PS1dE9 mice (APP mice) were used as animal models in the early stage of AD in this study. Acute/chronic chemical-kindling epilepsy models were established with pentylenetetrazol. Electroencephalogram and Racine scores were performed to assess seizures. Behavioral tests were used to assess cognition and emotion. Electrophysiology, western blot and immunofluorescence were performed to detect the alterations in synapses, GABAergic system components and NRG1-ErbB4 signaling. Furthermore, NRG1 was administrated intracerebroventricularly into APP mice and then its antiepileptic and cognitive effects were evaluated.

**Results:**

APP mice had increased susceptibility to epilepsy and resulting hippocampal synaptic damage and cognitive impairment. Electrophysiological analysis revealed decreased GABAergic transmission in the hippocampus. This abnormal GABAergic transmission involved a reduction in the number of parvalbumin interneurons (PV^+^ Ins) and decreased levels of GABA synthesis and transport. We also found impaired NRG1-ErbB4 signaling which mediated by PV^+^ Ins loss. And NRG1 administration could effectively reduce seizures and improve cognition in four-month-old APP mice.

**Conclusion:**

Our results indicated that abnormal GABAergic transmission mediated hippocampal hyperexcitability, further excitation/inhibition imbalance, and promoted epileptogenesis in the early stage of AD. Appropriate NRG1 administration could down-regulate seizure susceptibility and rescue cognitive function. Our study provided a potential direction for intervening in the co-morbidity of AD and epilepsy.

**Supplementary Information:**

The online version contains supplementary material available at 10.1186/s12964-024-01528-7.

## Introduction

Alzheimer’s disease (AD) is a neurodegenerative disease that lacks clinically effective treatments [1]. It is characterized by memory deterioration and other cognitive domain impairments. Notably, non-convulsive epileptiform activity is an under-recognized co-morbidity of AD [2, 3]. Compared with the general population, patients with early-onset AD have a significantly increased relative risk of unprovoked seizures, up to 87-fold [4]; even patients with late-onset AD have a greater rate of unprovoked seizures than individuals of comparable age [5]. Subclinical epileptiform activity may predate the emergence of clinical cognitive deficits in AD, which is associated with neuronal hyperexcitability [6]. Furthermore, abnormal neuronal activity can additively or secondarily exacerbate cognitive dysfunction, accelerating AD progression [7, 8]. Therefore, it is urgent to explore the mechanisms of epileptogenesis associated with AD and adopt targeted interventions.

The hippocampus plays a critical role in several cognitive domains. Neuronal degeneration and synaptic damage in this region may underlie the structural basis of progressive learning and memory deficits in AD. Research has shown that the hippocampus is particularly vulnerable in AD. Hippocampal neuronal overactivity precedes Aβ plaque formation and is one of the earliest phenomena in AD pathophysiology [9, 10]. Cellular hyperexcitability, hypersynchronized electrical activity, and extensive rewiring of hippocampal networks progressively amplify the excitation/inhibition (E/I) imbalance in AD. Epilepsy-like pathological changes, including ectopic expression of neuropeptide Y, sprouting of collateral mossy fibers crossing the granule cell layer, have been found in the hippocampus of AD mice [11].

Synaptic transmission is one of the most important modes of cell communication, and maintains homeostasis in the brain. Currently, research has attributed the co-morbidity of AD and epilepsy to brain hyperexcitability, primarily caused by abnormal elevations in glutamate-mediated excitatory synaptic transmission [12]. Little attention has been paid to impaired γ-aminobutyric acid (GABA)-mediated inhibitory synaptic transmission, which is often viewed as a compensatory alteration for glutamatergic damage. GABAergic abnormalities are strongly associated with hereditary and acquired epilepsy [13]. GABA antagonists that block glutamate decarboxylase (GAD) or the GABA_A_ receptor-chloride channel complex are proconvulsants; mutations in the genes encoding GABA receptor subunits or GABA transporters could cause various epileptic syndromes [14, 15]. At the same time, mounting evidence attests to the multifaceted impairment of the GABAergic system in AD. For example, GABA levels in cerebrospinal fluid and temporal cortex are significantly reduced in AD patients [16, 17]; GABAergic perisomatic synapses around plaques are diminished both in patients and transgenic mice [18]. Decreased GABA transmission in AD will lead to E/I imbalance [19]. The E/I balance is fundamental for maintaining normal physiological brain function. Abnormal inhibitory inputs could bring about crosstalk between AD and epilepsy during disease progression. However, it remains unclear whether the influence of altered synaptic GABAergic homeostasis on seizures and cognition in early AD.

Neuregulin 1 (NRG1) is a neurotrophic factor that acts by activating ErbB receptor tyrosine kinases. ErbB4 is the only autonomous NRG1-specific activated one [20]. NRG1-ErbB4 signaling participates in neuronal development and migration, axonal navigation, and synaptic plasticity [21–23]. Notably, NRG1 enhances evoked GABA release [24] and regulates neuronal activity through ErbB4 [25]. Further studies revealed that NRG1-ErbB4 signaling could exert an intrinsic inhibitory effect during epileptogenesis [26–28]. Seizure-upregulated NRG1-ErbB4 signaling would contribute to enhancing GABAergic transmission and promoting seizure termination. But this negative regulatory mechanism resulting from self-preservation is fragile and limited. Impaired NRG1-ErbB4 signaling exacerbates epilepsy development and seizure spread [26]. Furthermore, decreased NRG1 expression in the hippocampus of AD patients [29] may also provide a potential basis for epileptogenesis.

In this study, we investigated the impacts of epileptiform activities on cognitive function in the early stage of AD using 4-month-old APPswe/PS1dE9 mice (APP/PS1 mice) and the underlying mechanism of early AD susceptibility to epilepsy. Our results clarify that APP mice are prone to epilepsy. Impaired GABAergic transmission mediates upregulated epilepsy susceptibility, triggers hippocampal circuit cascade damage, and exacerbates cognitive impairment in APP/PS1 mice. Rescue of GABAergic inputs, such as regulating NRG1-ErbB4 signaling, may reduce the risk of epilepsy and intervene in the disease phenotype in APP/PS1 mice. Taken together, our study emphasizes the importance of impaired hippocampal GABAergic transmission in the development of AD, and provides a potential therapeutic direction for the co-morbidity of AD and epilepsy.

## Materials and methods

### Mice

The epileptic phenotype is most common in APP mice harboring the Swedish mutation [30]. We selected male APPswe/PSEN1dE9 (APP/PS1) transgenic mice and WT littermates from GemPharmatech. Mice were group housed under a 12 h light/dark cycle with food and water available ad libitum. All animal experimental procedures were approved by the Ethics Review Committee of Nanjing Drum Tower Hospital (No. 2023AE01038).

### PTZ kindling model

#### Surgery and acute kindling model preparation

##### WT vs. APP

Mice were anesthetized by isoflurane with an induction concentration of 5% and a maintenance concentration of 2–3%. They underwent surgical implantation of an electrode to register the cortical electrical activity. After a 3-day recovery period, the cortical electrical activity was digitized by the three-channel tethered systems (Pinnacle Technology Inc.) with a sampling rate of 800 Hz via Sirenia® Acquisition software (Pinnacle Technology Inc.). The electroencephalogram (EEG) baseline was recorded for 20–30 min. Mice received intraperitoneal injection with pentylenetetrazol (PTZ; Sigma-Aldrich, P6500) at chronological doses of 40 mg/kg, and then 20 mg/kg every 15 min [31] until they reached Racine V [32]. Signals were continuously recorded during PTZ-induced acute kindling.

##### APP + PBS vs. APP + NRG1

According to previous studies [27, 28], APP mice were anesthetized in a stereotaxic frame. They were injected with NRG1-β (100 µM in 2 µL) or vehicle (2 µL) into the right lateral ventricle (0.22 mm posterior to bregma, 1 mm lateral to midline and 2.5 mm below the skull surface). In detail, recombinant human NRG1-β/HRG1-β1 EGF domain proteins (R&D Systems, 396-HB-050) were reconstituted in sterile PBS containing 1% Bovine serum albumin (BSA). After 30 min of recovery, mice were given PTZ and their behavior was scored as above.

#### Chronic kindling model preparation

##### WT vs. APP

Mice were randomly divided into 4 groups: WT + SA, WT + PTZ, APP + SA and APP + PTZ. They were intraperitoneally injected with the same dose of normal saline (SA) or PTZ (30–35 mg/kg) daily. Their behavior was observed for 30 min after injection. The PTZ chronic kindling model was considered successfully established when mice reached Racine III-IV 3 times consecutively. Drug administration continued until 80% of mice in the APP + PTZ group were successfully modeled. Those unsuccessfully modeled mice were not included in subsequent experiments.

##### APP + PBS vs. APP + NRG1

In accordance with previous methods [33, 34], APP mice were anesthetized in a stereotaxic frame. They underwent surgical implantation of a guide cannula (RWD Instrument) into the right lateral ventricle. After a 7-day recovery period, mice were injected with NRG1-β (10 µM in 2 µL) or vehicle (2 µL) using a microinjection pump through an injection cannula (0.2 µL/min). They were then given the same dose of PTZ (30–35 mg/kg) daily and behavior was scored as above. Drug administration continued until 80% of mice in the APP + PBS group were successfully modeled. Those unsuccessfully modeled mice were not included in subsequent experiments.

### Behavioral tests

The open field test, Y-maze, novel object recognition (NOR) test, elevated plus-maze (EPM) and Morris water maze (MWM) were performed as previously described [19, 31, 35]. Mice were allowed to acclimatize to the environment for 1 h before all behavioral tests. After testing each mouse, the instruments were cleaned carefully with 75% alcohol before the next mouse was introduced.

#### Open field

The open field test was used to assess motor function and anxiety of mice. The instrument was a 48 cm × 48 cm × 36 cm box and divided into 16 identical squares. 4 corner squares were defined as the corner zone, and 4 middle squares were defined as the center zone. Mice were allowed to move freely for 10 min. We recorded and analyzed the moving speed and time in the corner/center zone.

#### Y-maze

The Y-maze test was used to assess the short-term spatial memory of mice. The instrument was a Y-shaped maze consisting of three identical arms (35 cm × 8 cm × 6 cm). Mice were allowed to move freely for 8 min. We recorded and analyzed the total number and order of arm entries. Spontaneous alternations were defined as continuous nonoverlapping entries into three different arms.

#### NOR

The NOR test was used to assess the short-term spatial memory of mice. The instrument was a 30 cm × 30 cm × 45 cm box. Mice were habituated to the testing box for three consecutive days before testing. During the familiarization session, mice were allowed to explore two identical objects freely for 10 min. After at least 30 min, one of the initial objects was removed and replaced with a new object in the original position. During the 5-min test session, the time that each mouse spent approaching the new object was recorded.

#### EPM

The EPM test was used to assess the anxiety-like behavior of mice. The instrument was a plus-shaped maze consisting of two open arms and two closed arms (30 cm × 5 cm × 15 cm), and it was elevated 50 cm above the ground. Each mouse was placed in the middle area and faced the open arm at the beginning. Mice were allowed to move freely for 5 min. We recorded and analyzed the number of open/closed arm entries and time spent in the open arm.

#### MWM

The MWM test was used to assess the spatial learning and memory of mice. The instrument consisted of a circular pool (120 cm diameter) and a 1-cm underwater escape platform. During the 5-day acquisition trial, mice were trained to locate the escape platform within 60 s. If the mouse couldn’t find the platform within the stipulated time, it was guided onto the platform and allowed to rest for 30 s. During the probe test (Day 6), mice were allowed to explore for 60 s with the platform removed. We recorded and analyzed the latency to reach the platform, mean swimming speed, number of platform crosses, time spent in the target quadrant and latency to reach the target quadrant with ANY-maze software (Stoelting Co.).

### Electrophysiology

Acute 300-µm hippocampal slices were prepared as described previously [19, 35, 36]. The slices were placed into the microelectrode array and perfused continuously with oxygenated artificial cerebrospinal fluid (ACSF; 2 mL/min, 32 ℃). Field excitatory postsynaptic potentials (fEPSPs) were generated from the CA1 stratum radiatum using the MEA-2100-60-System (Multi Channel Systems). The slope of fEPSPs was measured to analyze the input-output (I/O) relationships of synapses. In long-term potentiation (LTP) experiments, the stimulation intensity was 50% of the maximum evoked response and the stimulation frequency was 100 Hz (three trains, 1-s duration, 10-s interval time). The initial slopes of fEPSPs were normalized by the average value at baseline. The data were acquired with LTP-Director software and analyzed with LTP-Analyzer software.

For whole-cell patch clamp recordings, we observed the pyramidal neurons in CA1 using an upright microscope with a 40× water-immersion lens. Electrophysiological signals were recorded with MutiClamp 700B amplifiers, Digidata 1550B analog-to-digital converters and pClamp 10.7 software (Molecular Devices). We obtained stable whole-cell recordings (~ 20 MΩ) and then recorded basic electrophysiological properties.

Pyramidal neurons were held at -70 mV using voltage clamp. For miniature inhibitory postsynaptic currents (mIPSCs) recording, the external solution contained 20 µM CNQX, 50 µM D-APV and 1 µM TTX; the internal solution contained 130 mM CsCl, 1 mM MgCl_2_·6H_2_O, 0.2 mM EGTA, 10 mM HEPES, 10 mM Na_2_-phosphocreatine, 4 mM Mg-ATP, 0.3 mM Na-GTP and 5 mM QX-314. For miniature excitatory postsynaptic currents (mEPSCs) recording, the external solution contained 20 µM bicuculline and 1 µM TTX; the internal solution contained 125 mM potassium gluconate, 5 mM KCl, 1 mM MgCl_2_·6H_2_O, 0.2 mM EGTA, 10 mM HEPES, 10 mM Na_2_-phosphocreatine, 4 mM Mg-ATP and 0.3 mM Na-GTP.

### Western blot

Mouse hippocampal tissue lysates were prepared and subjected to western blot (WB) as previously described [36]. The primary antibodies used were as follows: mouse-anti β-actin (1:2000, Sigma, A5441), rabbit-anti ErbB4 (1:1000, Cell Signaling Technology, 4795), mouse-anti NRG1 (1:200, Santa Cruz Biotechnology, sc-393006), rabbit-anti GAD65 + 67 (1:2000, Abcam, ab11070), mouse-anti vGAT (1:1000, Synaptic Systems, 131011), rabbit-anti PSD-95 (1:1000, Abcam, ab18258), rabbit-anti Syn-1 (1:1000, Abcam, ab64581). The bands were visualized with the Gel-Pro system (Tanon) and quantified using ImageJ Fiji software.

### Immunofluorescence staining

After anesthesia, mice were transcardially perfused with PBS and 4% PFA. The brain samples were collected and fixed in 4% PFA for 24 h. The 20-µm brain sections were cut after dehydration. Immunofluorescence staining of brain sections was performed as previously described [36]. The primary antibodies used were as follows: mouse-anti Parvalbumin (1:200, Sigma, P3088), rabbit-anti Somatostatin (1:200, Millipore, MAB354), rabbit-anti ErbB4 (1:100, Cell Signaling Technology, 4795). Fluorescence images were captured by a confocal microscope (Olympus, FV1200) and analyzed using ImageJ Fiji software.

### TdT-mediated dUTP Nick End Labeling (TUNEL) assay

Brain slices were obtained as in Immunofluorescence staining and processed with a TUNEL brightgreen apoptosis detection kit (Vazyme Biotech Co.). Samples were permeabilized with 0.25% Triton X-100 for 20 min and blocked with 2% BSA for 2 h at room temperature. After being washed with PBS three times (10 min each), slices were covered with 1× Equilibration Buffer for 20 min and with the BrightGreen reaction mixture (enzyme solution + labeling solution) in the dark for 1 h, and were incubated with rabbit-anti NeuN (1:500, Abcam, ab177487) at 4℃ overnight. After being washed with PBS three times (10 min each), slices were incubated with secondary antibodies for 2 h, followed by DAPI staining (1:1000, Bioworld) for 20 min. Fluorescence images were captured by a fluorescence microscope (Olympus, DP80).

### Statistical analysis

All the results were represented as the means ± SEM and were analyzed using GraphPad Prism 8. Differences between groups were assessed, using the detailed statistical tests which were indicated in the figure legends. If the data were not normally distributed, we used nonparametric tests instead. Statistical significance was set at *p* < 0.05.

## Results

### Higher susceptibility to epilepsy of APP/PS1 mice

**Fig. 1:**
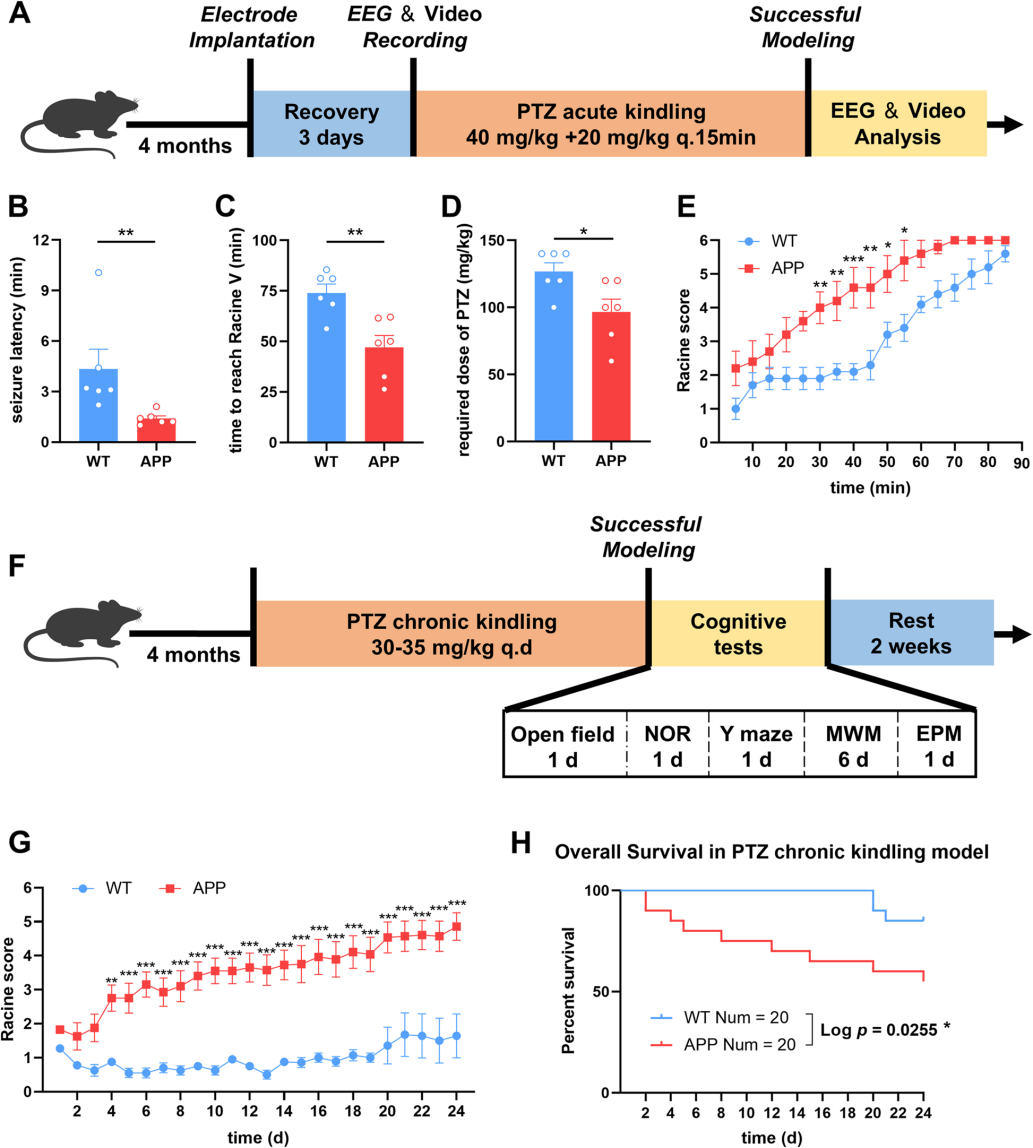
Higher susceptibility to epilepsy of APP/PS1 mice. **A** Experimental design of PTZ acute kindling model. **B**-**D** The latency of seizure phenotypes (**B**), time to reach Racine V (**C**) and required dose of PTZ (**D**) in 4-month-old WT and APP group. *N* = 6. **E** Maximal Racine score reached every 5 min within 90 min. *N* = 6. **F** Experimental design of PTZ chronic kindling model. **G** Maximal Racine score reached per day. *N* = 20. **H** Survival curve for PTZ chronic kindling model. *N* = 20. Data were shown as means ± SEM. Mann Whitney test for (**B**). Unpaired t test for (**C**, **D**). Two-way ANOVA for (**E** and **G**). Log-rank test for (**H**). *p < 0.05, ** *p* < 0.01, ***p < 0.001

To assess the seizure susceptibility in the early stage of AD, we established the acute and chronic PTZ chemical-kindling epilepsy models in 4-month-old APP and WT mice (Fig. 1A and F).

**Fig. 2:**
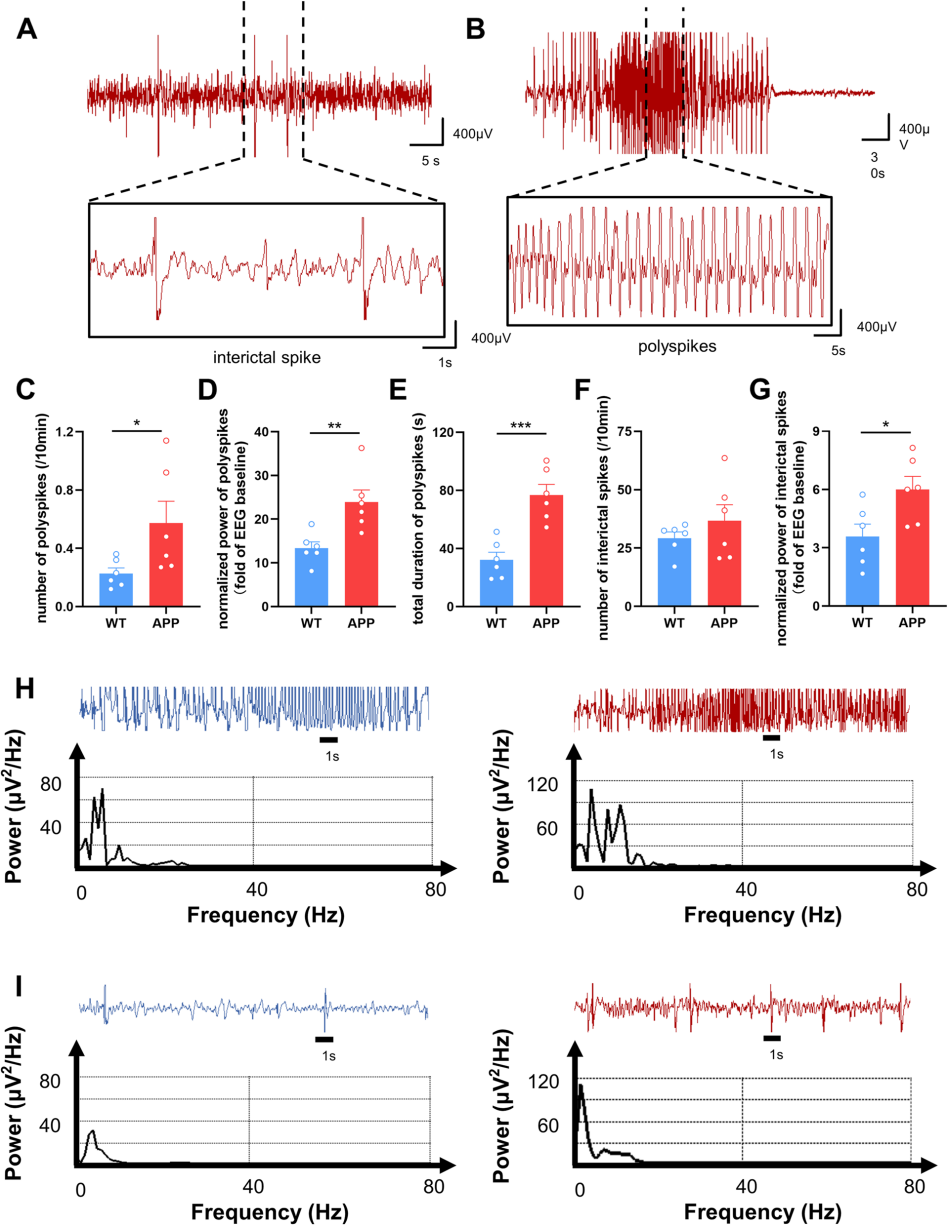
EEG characteristics in PTZ acute kindling model of APP/PS1 and WT mice. **A** and **B** Representative epileptiform activities, including interictal spike (**A**) and polyspikes (**B**). **C**-**E** The number (**C**), normalized power (**D**), and total duration (**E**) of polyspikes. *N* = 6. **F** and **G** The number (**F**) and normalized power (**G**) of interictal spikes. *N* = 6. **H** and **I** The typical spectrum analysis of polyspikes (**H**) and interictal spike (**I**) in WT (blue) and APP (red) mice. Data were shown as means ± SEM. Unpaired t test for (**C**-**G**). *p < 0.05, ** *p* < 0.01, ***p < 0.001

The acute modeling results revealed that APP mice had a shorter seizure latency, required less time and a smaller dose to reach the Racine V (Fig. 1B-D). It was easier for APP mice to achieve higher seizure levels during the same period (Fig. 1E). To more objectively evaluate seizures in mice, we analyzed the EEG characteristics in PTZ-induced acute kindling models. The results indicated that APP mice had more severe epileptiform activities, as indicated by more, stronger, longer-lasting polyspikes and stronger interictal spikes, than did WT mice (Fig. 2C-E and G). The spectrum analysis of polyspikes and interictal spikes also suggested the more severe epileptic conditions in APP mice (Fig. 2H-I). Besides, we compared seizure scores in APP mice at different ages. The required time of tonic-clonic seizures was negatively correlated with age (Fig. S1). Chronic modeling also revealed that APP mice more easily exhibited higher seizure levels, and had a higher mortality rate during PTZ kindling (Fig. 1G and H). Taken together, these results suggested that APP mice are more susceptible to epilepsy than WT.

### Seizures impair behavioral performances of APP/PS1 mice

**Fig. 3:**
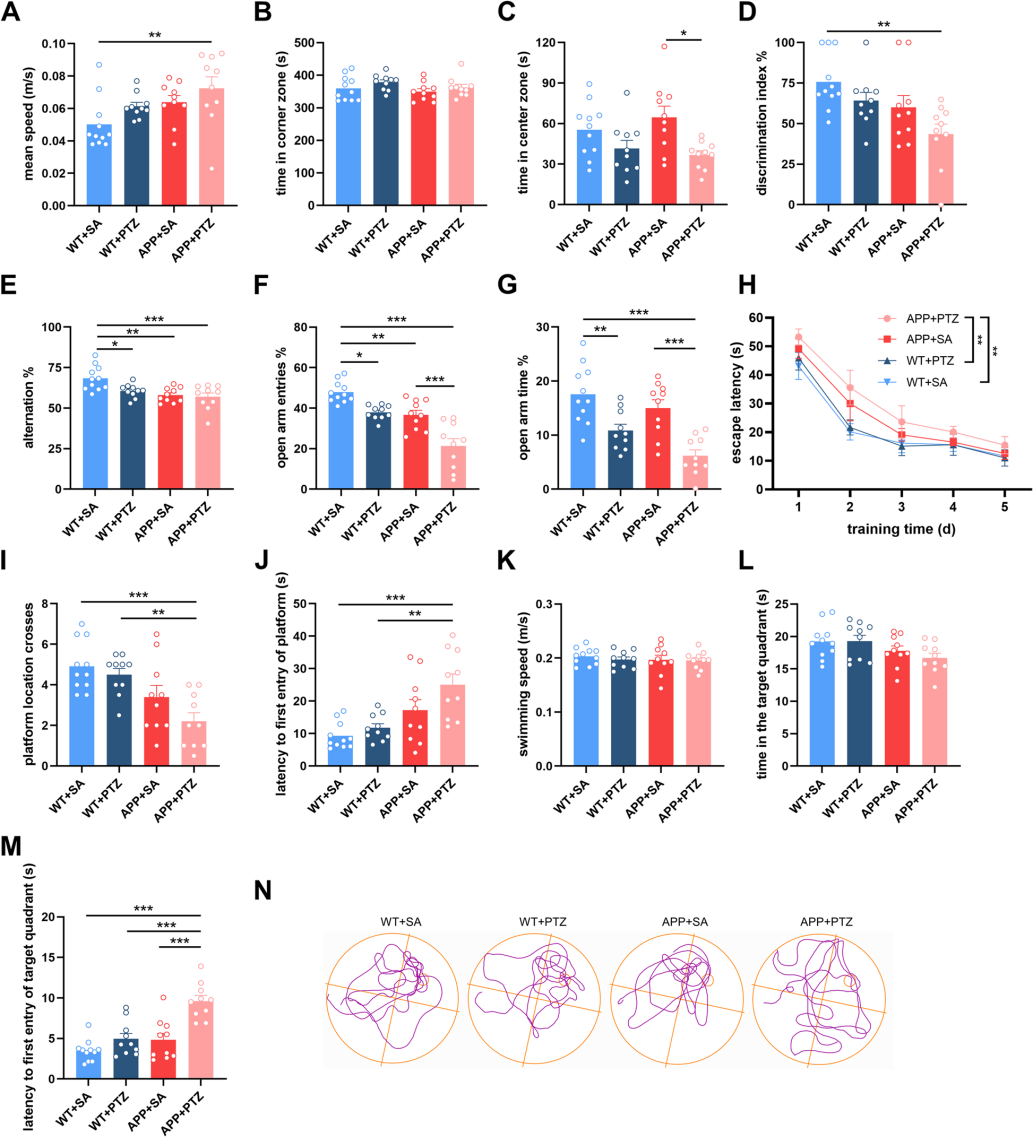
Seizures impair behavioral performances of APP/PS1 mice. **A**-**C** The mean speed (**A**), time in the corner zone (**B**) and time in the center zone (**C**) in the Open Field tests. **D** The percentage of time to explore new objects in the NOR tests. **E** The percentage of spontaneous alternation in the Y maze tests. **F** and **G** The percentage of entries into the open arm (**F**) and time in the open arm (**G**) in the EPM tests. **H** In the MWM tests, the escape latency in the acquisition trial. **I**-**M** In the MWM tests, the number of platform crosses (**I**), latency to platform (**J**), the mean swimming speed (**K**), time in the target quadrant (**L**) and latency to target quadrant (**M**) during the probe test. **N** Representative track images of mice in the probe test. *N* = 11, 10, 10, 10. Data were shown as means ± SEM. One-way ANOVA for (**C**-**G**, **I**-**M**). Kruskal-Wallis test for (**A** and **B**). Two-way ANOVA for (**H**). *p < 0.05, ** *p* < 0.01, ***p < 0.001

To detect the effects of seizures on phenotypes, PTZ-induced chronic kindling mice and SA-treated control mice were subjected to several behavioral tests. In the open field test, APP + PTZ mice moved faster than WT + SA (Fig. 3A) and spent less time in the center zone than APP + SA (Fig. 3C). Time spent in the corner zone was similar between the groups (Fig. 3B). These results suggested that APP + PTZ mice might have anxiety. We further conducted the elevated plus-maze test. Consistently, PTZ-induced chronic kindling mice tended to avoid entering or staying in the open arm compare with SA control (Fig. 3F and G). In addition, APP + PTZ mice showed fewer discrimination indices in NOR test (Fig. 3D) and made fewer spontaneous alterations in the Y-maze test (Fig. 3E) than WT + SA, which suggested a seizure-induced impairment of short-term spatial memory. In the acquisition trial of the MWM test, APP + PTZ mice exhibited the longest latency to reach the platform (Fig. 3H). During the probe test, the number of platform location crosses of APP + PTZ mice was significantly decreased (Fig. 3I), and latency to first entry to the platform and the target quadrant both increased (Fig. 3J and M). There was no significant difference in mean speed or time spent in the target quadrant between the groups (Fig. 3K and L). These results indicated seizure-induced impairment of spatial learning and memory in APP + PTZ mice.

### Seizures induce synaptic loss and worsen neuronal death in the hippocampus of APP/PS1 mice

**Fig. 4:**
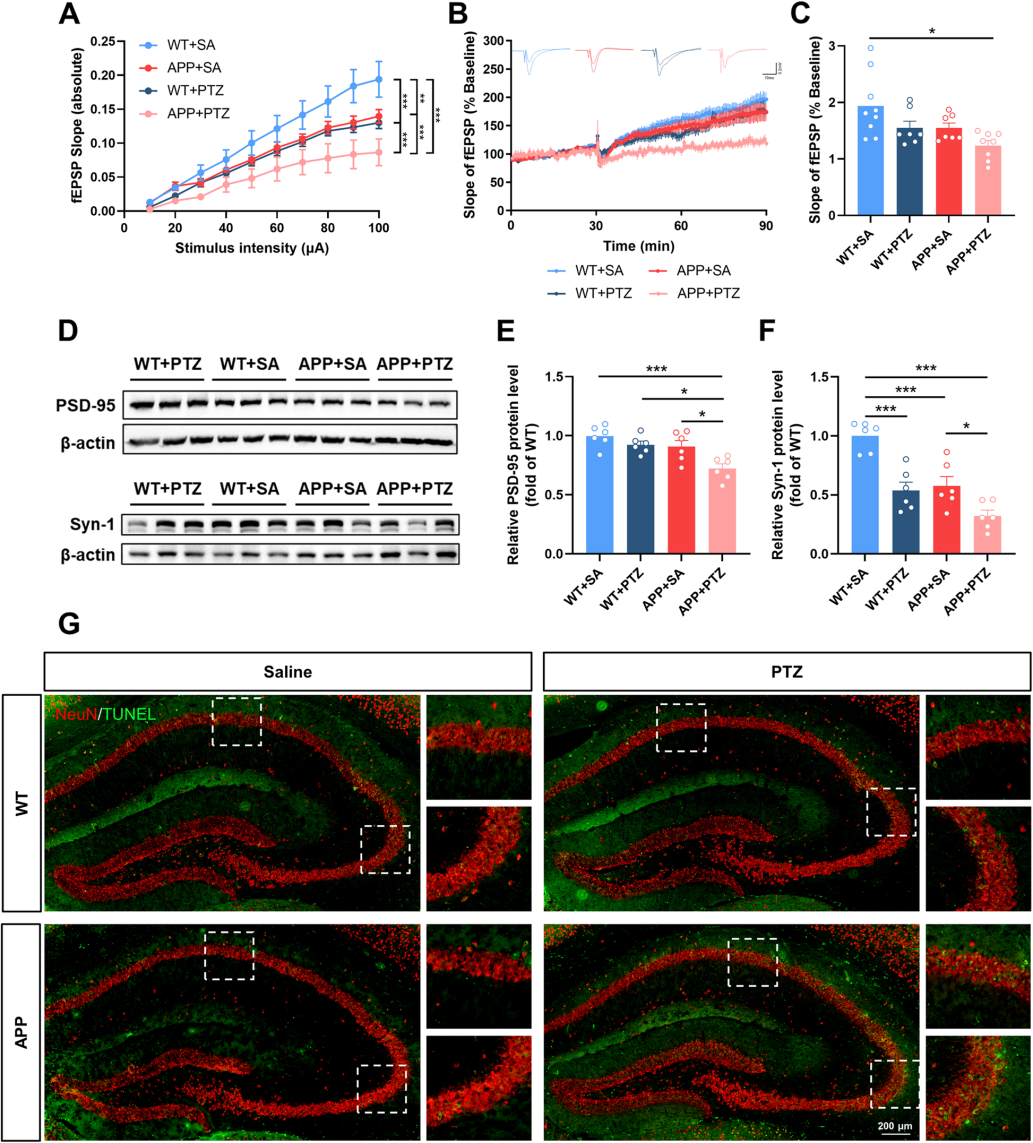
Seizures induce synaptic loss and worsen neuronal death in the hippocampus of APP/PS1 mice. **A** The I-O curve of hippocampal CA1. *N* = 3–4 mice per group, *n* = 7–9 slices per mouse. **B** and **C** LTP induced by high-frequency stimulation was evaluated in hippocampal CA1. *N* = 3–4 mice per group, *n* = 7–9 slices per mouse. **D**-**F** Western blot and quantitative analysis for synapse-associated proteins in the hippocampus, including PSD-95 (**E**) and Syn-1 (**F**). *N* = 6. **G** Representative images of neuronal apoptosis in the hippocampal CA1 and CA3 regions. Scale bar, 200 μm. Data were shown as means ± SEM. Two-way ANOVA for (**A**, **C**, **E** and **F**). *p < 0.05, ***p < 0.001

We further detected the effects of seizures on synapses, especially synaptic plasticity and relevant protein expression, in the hippocampus of APP mice. Hippocampal synaptic transmission and LTP induction in the APP mice receiving PTZ injection were significantly impaired (Fig. 4A-C). The protein levels of PSD-95 and Syn-1 were significantly decreased in APP + PTZ mice compared with APP + SA mice (Fig. 4D-F). These data suggested that seizures could cause worse synaptic structure and function in APP mice.

Furthermore, we used TUNEL assay to detect neuronal damage in each group. There were more TUNEL-positive neurons in the hippocampal CA1 and CA3 regions of APP + PTZ mice compared with APP + SA (Fig. 4G). These results suggested increased neuronal damage in PTZ-kindling APP mice, which may disrupt of neural circuit integrity and further promote E/I imbalance.

### Impaired GABAergic inputs in the hippocampus of APP/PS1 mice

**Fig. 5:**
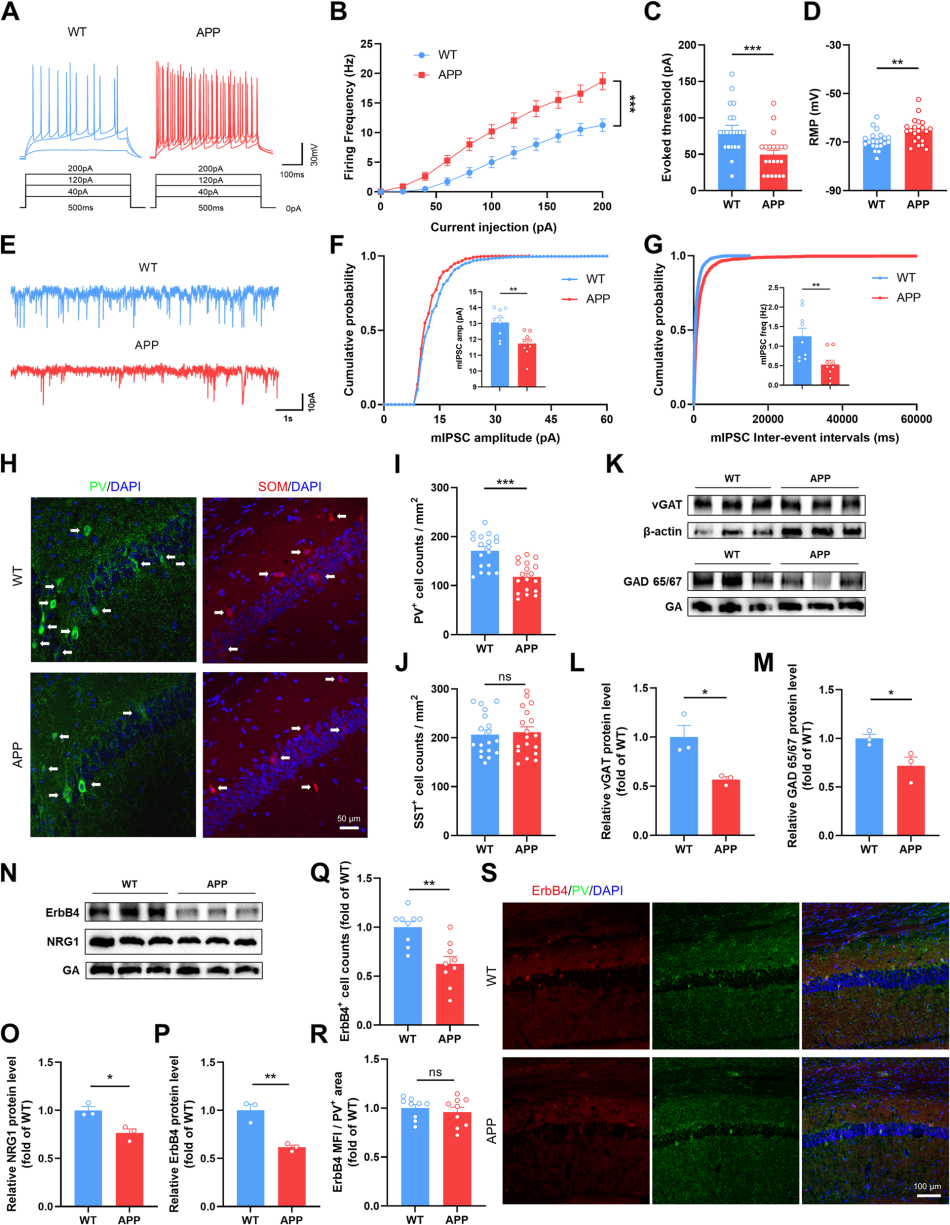
Impaired GABAergic inputs in the hippocampus of APP/PS1 mice. **A** Representative AP traces of evoked spikes. **B** Average AP firing frequency of CA1 pyramidal neurons in response to 0- to 200-pA depolarizing current steps. *N* = 3 mice per group, *n* = 6–7 neurons per mouse. **C** Evoked threshold of CA1 pyramidal neurons of APP and WT mice. *N* = 3 mice per group, *n* = 6–7 neurons per mouse. **D** Rest membrane potential of CA1 pyramidal neurons of APP and WT mice. *N* = 3 mice per group, *n* = 6–7 neurons per mouse. **E** Representative traces of mIPSC recordings in hippocampal CA1 region. **F**, **G** Mean mIPSC amplitude (**F**) and frequency (**G**) in CA1 pyramidal neurons. *N* = 3 mice per group, *n* = 3–5 neurons per mouse. **H** Representative fluorescence images showing the PV^+^ Ins (green) and SOM^+^ Ins (red) in the hippocampal CA1 region of APP and WT mice. Scale bar, 50 μm. **I** Comparable number of PV^+^ Ins in the hippocampal CA1 region of APP and WT mice. *N* = 3 mice per group, *n* = 6 (average of 12 slices) per mouse. **J** Comparable number of SOM^+^ Ins in the hippocampal CA1 region of APP and WT mice. *N* = 3 mice per group, *n* = 6 (average of 12 slices) per mouse. **K**-**P** Western blot and quantitative analysis for vGAT (**L**), GAD65/67 (**M**), NRG1 (**O**) and ErbB4 (**P**). *N* = 3. **Q**, **R** Quantitative analysis for number of ErbB4^+^ cells per unit area (**Q**), and mean fluorescent intensity of ErbB4 particularly in PV^+^ Ins (**R**). *N* = 3 mice per group, *n* = 3 (average of 9 slices, 72–81 cells) per mouse. **S** Representative fluorescence images showing the PV^+^ Ins (green) and ErbB4 (red) in the hippocampal CA1 region of APP and WT mice. Scale bar, 50 μm. Data were shown as means ± SEM. Two-way ANOVA for (**B**). Mann Whitney test for (**C**). Unpaired t test for (**D**, **F**, **G**, **I**, **J**, **L**, **M**, **O**-**R**). *p < 0.05, ** *p* < 0.01, ***p < 0.001

We measured the action potentials (APs) of hippocampal CA1 pyramidal neurons through whole-cell patch clamp recordings. The results showed that the firing frequency and resting membrane potential (RMP) were significantly increased and that the evoked threshold was decreased in APP group (Fig. 5A-D), indicating hyperexcitability of the cells.

Increased excitatory inputs or/and decreased inhibitory inputs result in hyperexcitability. To investigate whether glutamatergic or GABAergic transmission is altered, we measured mEPSCs and mIPSCs in hippocampal CA1 pyramidal neurons. The amplitude and frequency of mEPSCs were similar between the groups (Fig. S2A-C), indicating normal glutamatergic transmission. However, both amplitude and frequency of mIPSCs were significantly decreased in APP mice (Fig. 5E-G). These results suggested impaired GABAergic neurotransmission in 4-month-old APP, which mediates hippocampal E/I imbalance and pyramidal neuronal hyperexcitability.

Parvalbumin interneurons (PV^+^ Ins) and somatostatin interneurons (SOM^+^ Ins) are the two main inhibitory interneurons of the brain that control pyramidal neuron activity and regulate E/I balance. We examined the number of PV^+^ and SOM^+^ cells in hippocampus by immunofluorescence. The results showed that the number of PV^+^ Ins was significantly decreased in the CA1 and DG regions of APP mice compared with WT (Fig. 5H and I; Fig. S3A-C). Moreover, there was no significant difference in the number of SOM^+^ Ins (Fig. 5H and J; Fig. S3D-F). The loss of inhibitory interneurons is responsible for impaired GABA release and decreased inhibition of excitatory neurons, which facilitates the initiation and propagation of seizures [37]. GAD 65/67 and vesicular GABA amino acid transporter (vGAT) are proteins responsible for GABA synthesis and transport respectively. Their expression levels were significantly decreased as well (Fig. 5K-M).

As mentioned above, NRG1-ErbB4 signaling is crucial for maintaining the E/I balance, especially for the GABAergic system. Hence, we detected the protein expression levels of NRG1 and ErbB4. The WB results suggested that NRG1-ErbB4 signaling was significantly impaired in APP hippocampus (Fig. 5N-P). ErbB4 is expressed primarily in PV^+^ Ins [38]. Consistent with the WB results, the number of ErbB4^+^ cells per unit area was significantly decreased. However, we found no significant changes in ErbB4 expression in single PV^+^ Ins by immunofluorescence (Fig. 5Q-S). In other words, the decrease in hippocampal ErbB4 expression in APP mice was mainly due to a decrease in the number of PV^+^ Ins.

### Administration of NRG1 down-regulates seizure susceptibility and rescues cognitive function of APP/PS1 mice

**Fig. 6:**
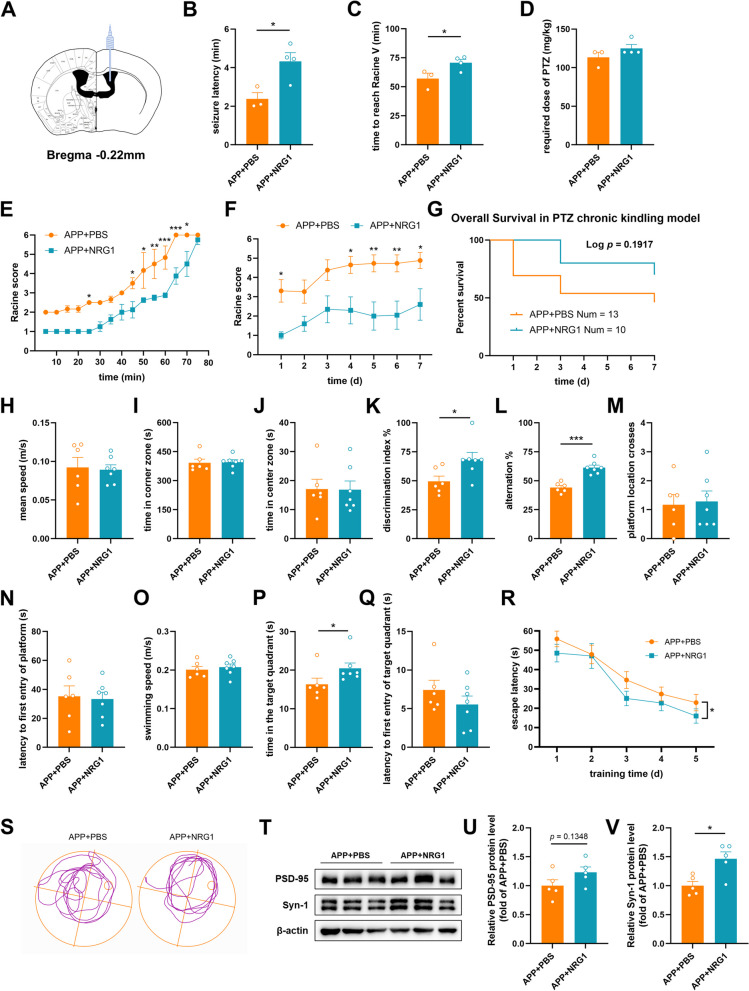
Administration of NRG1 down-regulates seizure susceptibility and rescues cognitive function of APP/PS1 mice. **A** Schematic diagram of intracerebroventricular injection. **B**-**D** The latency of seizure phenotypes (**B**), time to reach Racine V (**C**) and required dose of PTZ (**D**) in APP + PBS and APP + NRG1 acute kindling models. *N* = 3, 4. **E** Maximal Racine score reached every 5 min within 75 min. *N* = 3, 4. **F** Maximal Racine score reached per day. *N* = 13, 10. **G** Survival curve for PTZ chronic kindling model. *N* = 13, 10. **H**-**J** The mean speed (**H**), time in the corner zone (**I**) and time in the center zone (**J**) were recorded in the Open Field tests. *N* = 6, 7. **K **The percentage of time to explore new objects was recorded in the NOR tests. *N* = 6, 7. **L** The percentage of spontaneous alternation was recorded in the Y maze tests. *N* = 6, 7. **M**-**Q** In the MWM tests, the number of platform crosses (**M**), latency to platform (**N**), the mean swimming speed (**O**), time in the target quadrant (**P**) and latency to target quadrant (**Q**) were recorded during the probe test. **R** In the MWM tests, the escape latency was recorded in the acquisition trial. **S** Representative track images of mice in the probe test. *N* = 6, 7. **T**-**V** Western blot and quantitative analysis for synapse-associated proteins in the hippocampus, including PSD-95 (**U**) and Syn-1 (**V**). *N* = 5. Data were shown as means ± SEM. Two-way ANOVA for (**E**, **F**, **R**). Mann Whitney test for (**D**, **P**). Unpaired t test for (**B**, **C**, **H**-**O**, **Q**, **U**, **V**). Log-rank test for (**G**). *p < 0.05, ** *p* < 0.01, ***p < 0.001

NRG1 has been shown to repress limbic epileptogenesis in a rat kindling model [26]. We sought to explore whether the administration of exogenous NRG1 could alter epilepsy susceptibility in APP mice (Fig. 6A). Intracerebroventricular infusion of NRG1 significantly prolonged seizure latency and time to reach Racine V (Fig. 6B-C), and down-regulated seizure severity (Fig. 6E) in the acute kindling model. NRG1 administration also reduced the seizure levels in the chronic kindling model (Fig. 6F). Compared with those in the PBS-treated group, the mortality in APP + NRG1 group showed a downward trend (Fig. 6G).

To further explore the effects of NRG1 on cognition, we detected the behavioral performance of the APP + NRG1 group compared with that of the APP + PBS group. After chronic seizure kindling, APP + NRG1 mice showed no significant alterations in motor function or mood (Fig. 6H-J). Their performance in short-term spatial memory tests was significantly improved (Fig. 6K-L). Consistent with the findings of Ryu et al. [39], the APP + NRG1 mice in our study performed better in the acquisition trial of MWM test (Fig. 6R) and spent more time in the target quadrant during the probe test (Fig. 6P). In addition, we detected synaptic protein expression levels. PSD-95 expression had an upward trend and Syn-1 expression was significantly increased in APP + NRG1 mice (Fig. 6T-V). These data suggested that NRG1 administration down-regulated seizure susceptibility and partially rescued the cognitive function of 4-month-old APP mice.

## Discussion

In this study, we demonstrated that epilepsy susceptibility was upregulated in 4-month-old APP/PS1 mice compared with their WT littermates. And these PTZ-induced chronic kindling APP mice exhibited short-term spatial memory impairment, long-term learning and memory impairment and anxiety-like behaviour. In addition, seizures caused by PTZ, a GABA receptor antagonist, induced synaptic loss and neuronal death in the hippocampus of APP mice. Pyramidal neuronal hyperexcitability might underlie epileptogenesis in mouse brain. We found that hippocampal GABAergic but not glutamatergic transmission was abnormal in 4-month-old APP mice. Decreased GABAergic neurotransmission was reflected in several ways. Our results revealed reduced number of PV^+^ Ins, decreased expression of GABA synthesis and transport proteins, and impaired NRG1-ErbB4 signaling. Intracerebroventricular NRG1 administration could consequently down-regulated epilepsy susceptibility and ameliorated seizure-induced cognitive impairment.

Subclinical epilepsy is a co-morbidity of AD that should not be ignored [3]. It is estimated that 10-22% of patients with AD will develop unprovoked seizures [7]. People carrying mutations associated with familial Alzheimer’s disease, especially *PSEN1* mutations, are more commonly affected by seizures and myoclonus [40]. Similarly, spontaneous seizures and spike-wave discharges have been observed in AD-related animal models [30, 41]. Taking the classic APP/PS1 mice as objects, our results showed that they had lower seizure-inducing thresholds and more severe seizure phenotypes compared with age-matched WT. In addition, there is evidence of a positive correlation between seizures and Aβ deposition in both AD patients and mice [30, 42]. Aβ can increase excitatory neuronal activity [43], produce spontaneous neuronal firing [44] and enhance seizure events in the hippocampus [45]. Aβ pathology progresses gradually with age. Consistently, our results revealed that older APP mice required less time to achieve grand mal seizures.

Increased seizures will accelerate AD disease progression. Compared with the general population, people with epilepsy are three times more likely to develop dementia [46]. A cross-sectional study showed that 59% of patients with late-onset epilepsy of unknown etiology had mild cognitive impairment at diagnosis [47]. Epileptiform activities can exacerbate cognitive impairment, accelerate symptom progression and cause more severe neuronal loss in AD [7, 48]. Seizures also can promote the production of synaptotoxic neurodegenerative proteins, which in turn fosters extra Aβ deposition and facilitates neuronal damage, worsening neurodegeneration and initiating a vicious circle [49, 50]. Our results demonstrated that the negative impact of increased epileptic activity on cognitive function in APP mice, as well as synaptic and neuronal loss.

E/I imbalance plays a critical role in the pathogenesis of AD and epilepsy co-morbidity. Previous literature suggested that an impaired glutamate-glutamine cycle might underpin the epilepsy susceptibility in AD [51]. AD pathology involves disrupted glutamate reuptake by astrocytes [52], mutations in genes encoding NMDA and AMPA glutamate receptor subunits and the resulting altered ion channel activity [53]. These may disrupt glutamatergic homeostasis and promote epilepsy. In contrast, although multiple studies supported that aberrant GABAergic transmission was at the intersection of AD and epilepsy [14, 54], this abnormality was initially thought to be a compensatory alteration for glutamatergic damage [11]. Our results showed impaired GABAergic transmission but not glutamatergic transmission in the hippocampus of 4-month-old APP mice, suggesting that impairment of inhibitory synaptic transmission in the hippocampus occurred earlier.

Inhibitory GABAergic interneurons play an important role in regulating the activity of excitatory neurons and maintaining normal neural circuit activity through neurotransmitter release of GABA [55, 56]. As one of the major inhibitory interneurons in the hippocampus, the loss of PV^+^ Ins would result in an increase of local excitability. Furthermore, a reduced number or dysfunction of PV^+^ Ins can lead to deficits in working memory and executive function, and is strongly associated with abnormalities in the hippocampus-related networks in AD [57, 58]. Our results showed the number of PV^+^ Ins decreased specifically and significantly in 4-month-old APP mice. The global reduction of the GABAergic system components in the AD brain has been increasingly confirmed. Consistent with the findings of a recent meta-analysis [59], our results showed decreased expression of GAD65/67 and GABA transporters, leading to blockade of GABA production and transport. Taken together, extensive GABA-mediated inhibitory synaptic transmission abnormalities contribute to E/I imbalance and further epileptogenesis in APP mice.

NRG1-ErbB4 signaling is closely related to neurotransmission and synaptic plasticity [60]. NRG1 is abundantly distributed in the hippocampus of adult mice [61]. Its receptor, ErbB4, is highly expressed in GABAergic neurons, particularly PV^+^ Ins [38]. Exogenous NRG1 could suppress the induction of LTP at Schaffer collateral-CA1 synapses [62]. In detail, NRG1 increases the release of GABA from presynaptic terminals in CA1 pyramidal cells. Specific ablation of ErbB4 in PV^+^ Ins will eliminate the effects of NRG1 [60]. Mounting evidence has confirmed the negative regulation of epilepsy by NRG1-ErbB4 signaling [26–28, 33]. ErbB4 variants or exonic deletions are associated with genetic generalized epilepsy [63, 64] and early myoclonic encephalopathy [65]. PV^+^ Ins were further found to be responsible for NRG1-ErbB4 signaling to realize the antiepileptic effect [26]. Our results demonstrated that a reduction in the number of PV^+^ Ins in APP mice indirectly promoted epileptogenesis by impairing the NRG1-ErbB4 signaling. Furthermore, we confirmed the inhibitory effect of NRG1 on epilepsy in APP mice via intracerebroventricular infusion.

Besides, we focused on the resulting cognitive impacts of NRG1 by reducing seizures. Previous studies on the direct influence of NRG1 administration on cognitive function in AD remain controversial. On the one hand, NRG1-ErbB4 signaling could exert protective effects against cognitive deficits in AD models. NRG1 attenuated impairments in learning and memory and reduction in the number of dendritic spines [39]. A single local injection of NRG1 in the CA2 region could restore the PV/perineuronal net level and social memory [66]. On the other hand, ErbB4 inhibition might be beneficial for AD. NRG1-ErbB4 signaling could activate the mTOR pathway [67] and thus promote Tau hyperphosphorylation [68]. And ErbB4 might directly activate the JNK/Tau axis [69]. Blocking hippocampal NRG1/ErbB4 signaling or ablating ErbB4 attenuated synaptic and cognitive deficits [70, 71]. In addition, while NRG1 expression was decreased in the hippocampus [29], the cerebrospinal fluid and plasma concentrations of NRG1 were increased and inversely correlated with cognitive scores in AD patients [72, 73]. An inverted U curve illustrated the relationship between NRG1-ErbB4 level/activity and behavioral performance. Either too much or too little NRG1/ErbB4 signaling is detrimental [21]. It’s extraordinarily important to clarify the ‘appropriate’ range of NRG1/ErbB4 in the AD brain. To balance epilepsy and additional potential cognitive impairment, we must further explore the appropriate timing and dose of NRG1 administration according to the pathophysiologic state of AD.

Our previous study demonstrated decreased GABA transmission in the early stage of AD [19]. Accordingly, we selected PTZ, a GABA receptor antagonist, to further disrupt GABAergic homeostasis. On this basis, we induced epileptogenesis and investigated whether altered GABAergic homeostasis will cause cognitive decline. But there are some limitations should be acknowledged in this study. The specific mechanisms by which GABAergic imbalance mediates cognitive decline have not been explored in depth. Researchers recognize the importance of abnormal synaptic transmission in the pathogenesis and development of AD constantly. Seizures mediated by decreased GABAergic inputs may be just one cause of early cognitive impairments. In addition, the activity of remaining PV^+^ Ins in the hippocampus needs to be further detected. PV^+^ Ins in the prefrontal lobe showed increased evoked threshold and decreased frequency of APs in young APP mice [19].

In summary, this study demonstrated the susceptibility to epilepsy and cognitive impairments of mice with AD in the early stage, and further uncovered the underlying mechanism by which decreased GABAergic transmission induce E/I imbalance and even epileptogenesis. In addition, this study suggested the important role of NRG1-ErbB4 signaling in the co-morbidity of AD and epilepsy. These findings provide potential targets for delaying the disease process and reducing epilepsy in the early stages of AD.

### Supplementary Information

**Supplementary Material 1.**

**Supplementary Material 2.**

## Data Availability

No datasets were generated or analysed during the current study.
