# Supplementary material for: Impairments of GABAergic transmission in hippocampus mediate increased susceptibility of epilepsy in the early stage of Alzheimer’s disease
Source: Cell Commun Signal. 2024 Feb 22;22:147. doi: 10.1186/s12964-024-01528-7 (PMC10885444; doi:10.1186/s12964-024-01528-7)
Supplement: Supplementary file 1 — Supplementary Material 1. [file 12964_2024_1528_MOESM1_ESM.docx]

**
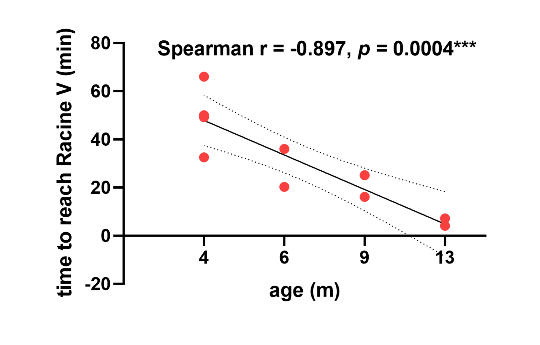
**

**Fig. S1** The required time to reach Racine V in APP mice is negatively correlated with age (Spearman *r* = -0.897, *p* = 0.0004).


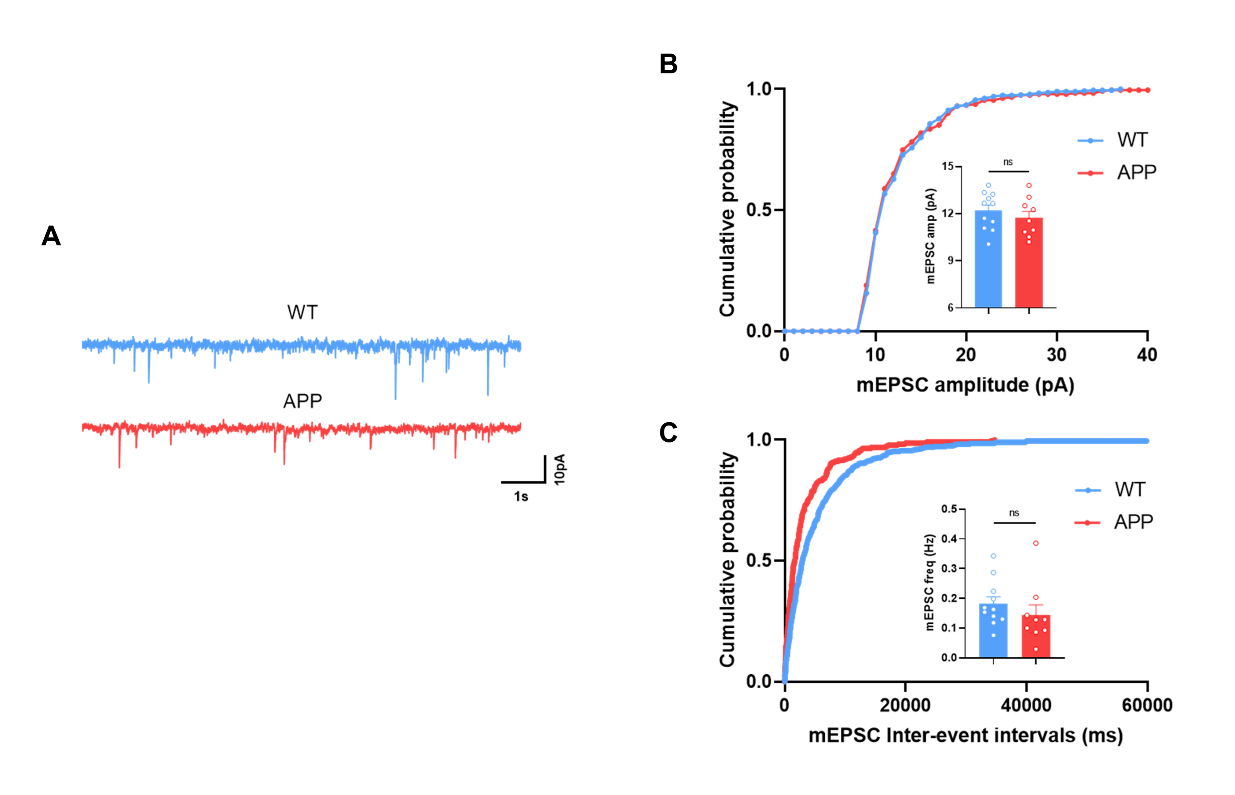


**Fig. S2** mEPSC attributes are similar between APP and WT mice. (A) Representative traces of mEPSC recordings in hippocampal CA1 region. (B-C) Mean mEPSC amplitude (F) and frequency (G) in CA1 pyramidal neurons. N = 3 mice per group, n = 3-5 neurons per mouse. Data were shown as means ± SEM. Unpaired t test for (B-C). ns, no significant.


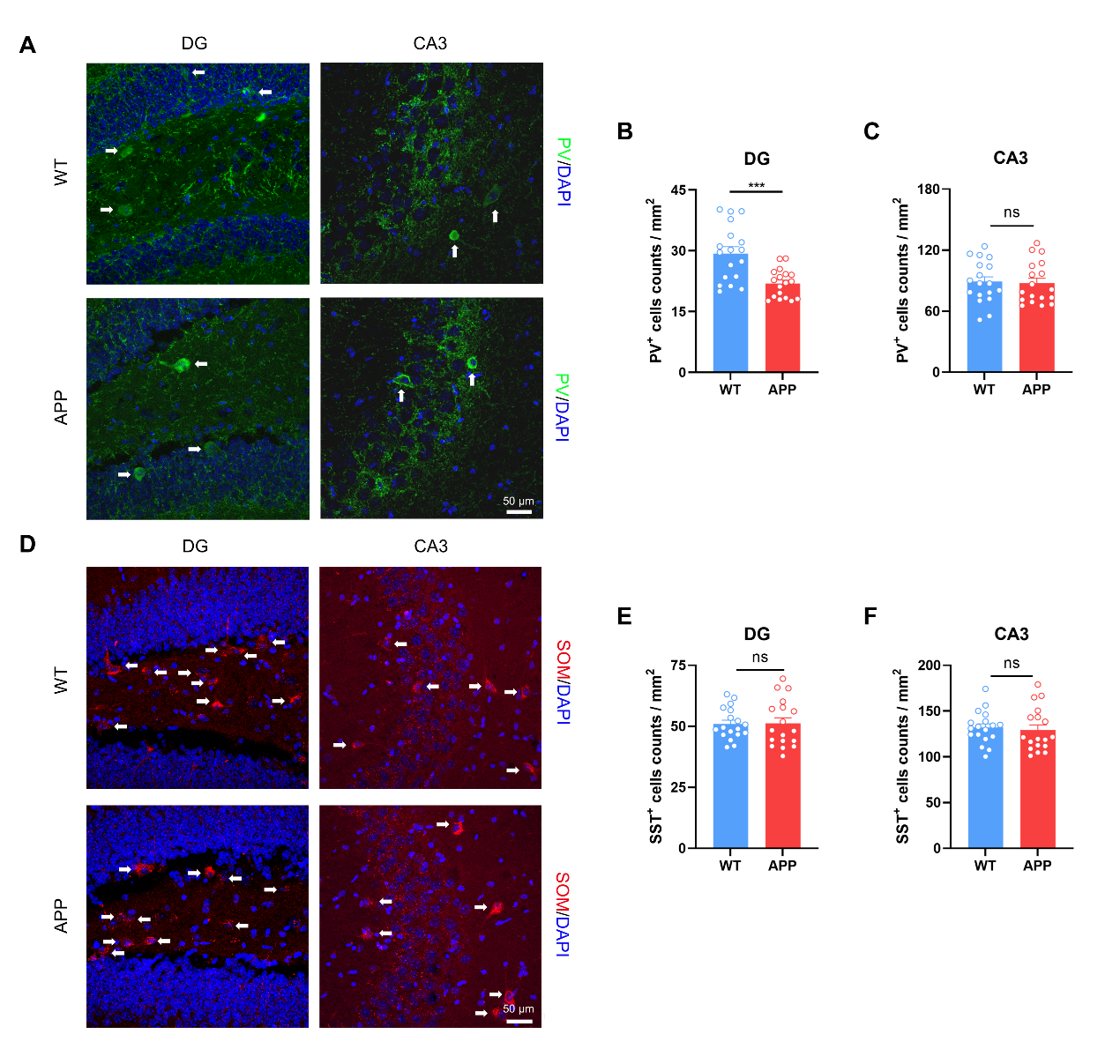


**Fig. S3** Statistics on the distribution of PV^+^ and SOM^+^ Ins in the DG and CA3 regions of the hippocampus. (A) Representative fluorescence images showing the PV^+^ Ins (green) in the hippocampal DG and CA3 regions of APP and WT mice. Scale bar, 50 μm. (B-C) Comparable number of PV^+^ Ins in the hippocampal DG (B; *t* = 4.085, *p* = 0.0003) and CA3 (C) regions of APP and WT mice. N = 3 mice per group, n = 6 (average of 12 slices) per mouse. (D) Representative fluorescence images showing the SOM^+^ Ins (red) in the hippocampal DG and CA3 regions of APP and WT mice. Scale bar, 50 μm. (E-F) Comparable number of SOM^+^ Ins in the hippocampal DG (E) and CA3 (F) regions of APP and WT mice. N = 3 mice per group, n = 6 (average of 12 slices) per mouse. Data were shown as means ± SEM. Unpaired t test for (B-C, E-F). ****p* < 0.001; ns, no significant.
