# Supplementary figures and images for: Impairments of GABAergic transmission in hippocampus mediate increased susceptibility of epilepsy in the early stage of Alzheimer’s disease
Source: Cell Commun Signal. 2024 Feb 22;22:147. doi: 10.1186/s12964-024-01528-7 (PMC10885444; doi:10.1186/s12964-024-01528-7)

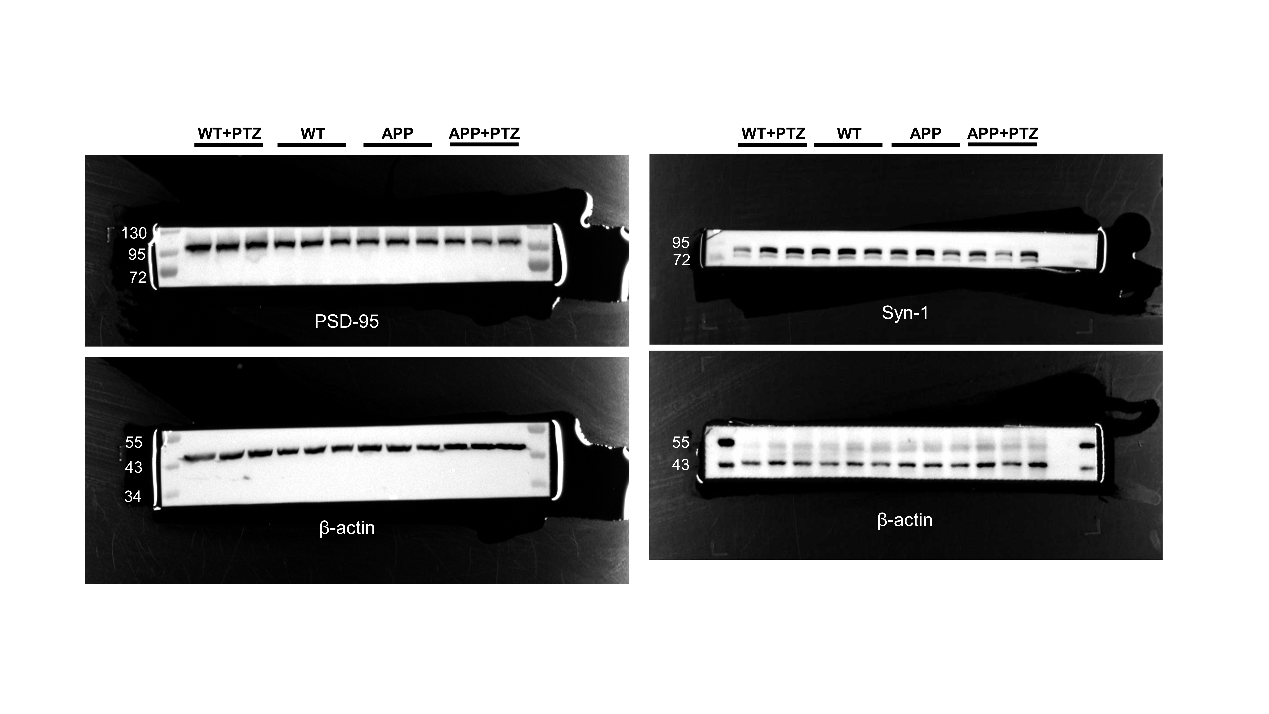


**A**


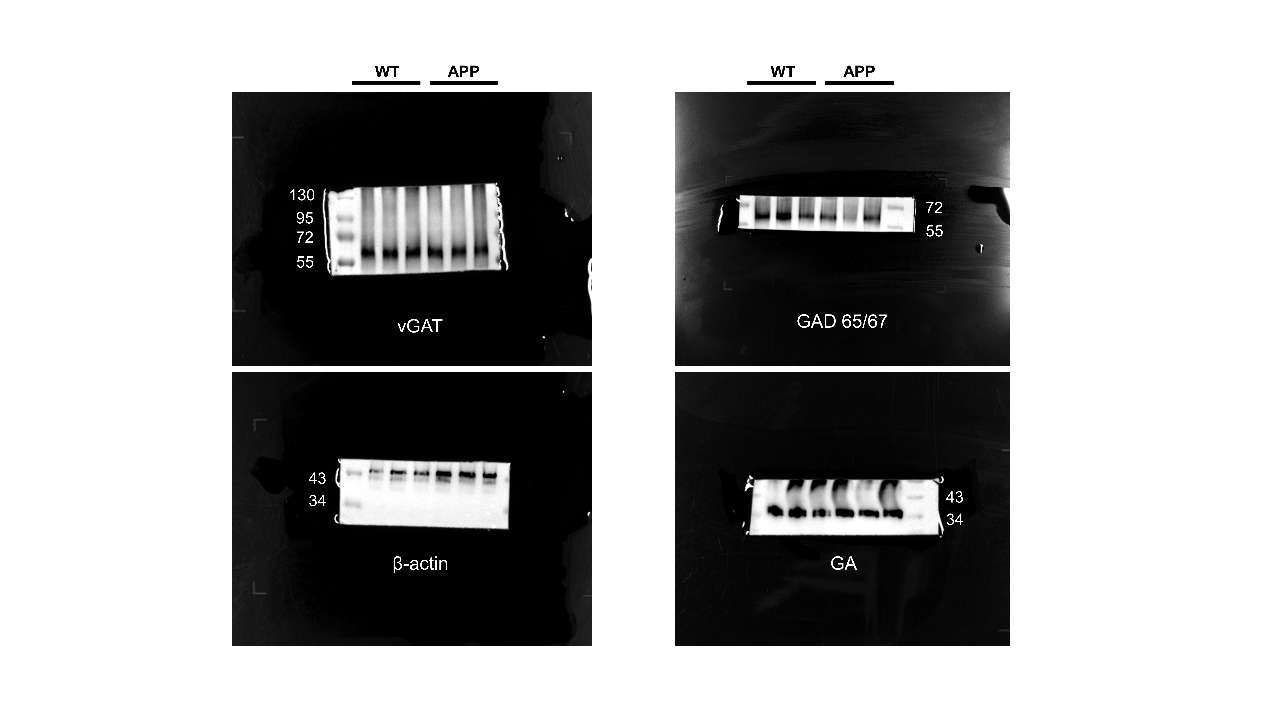


**B**


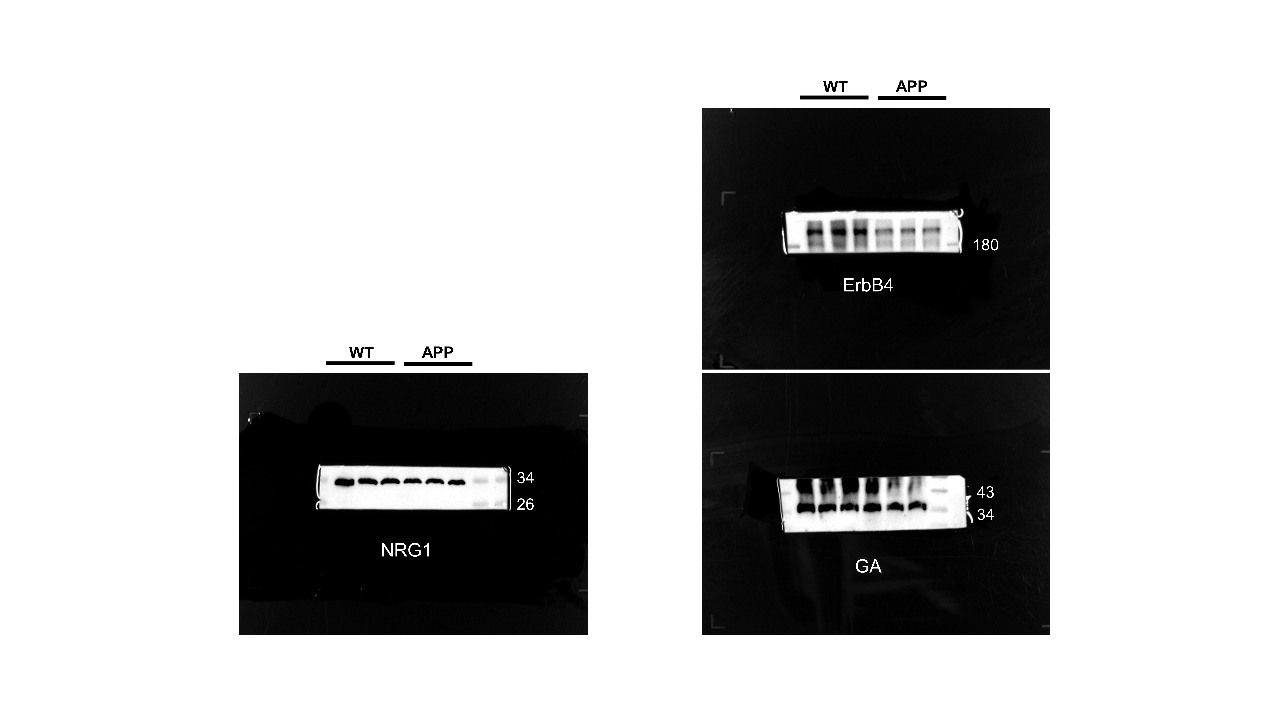


**C**


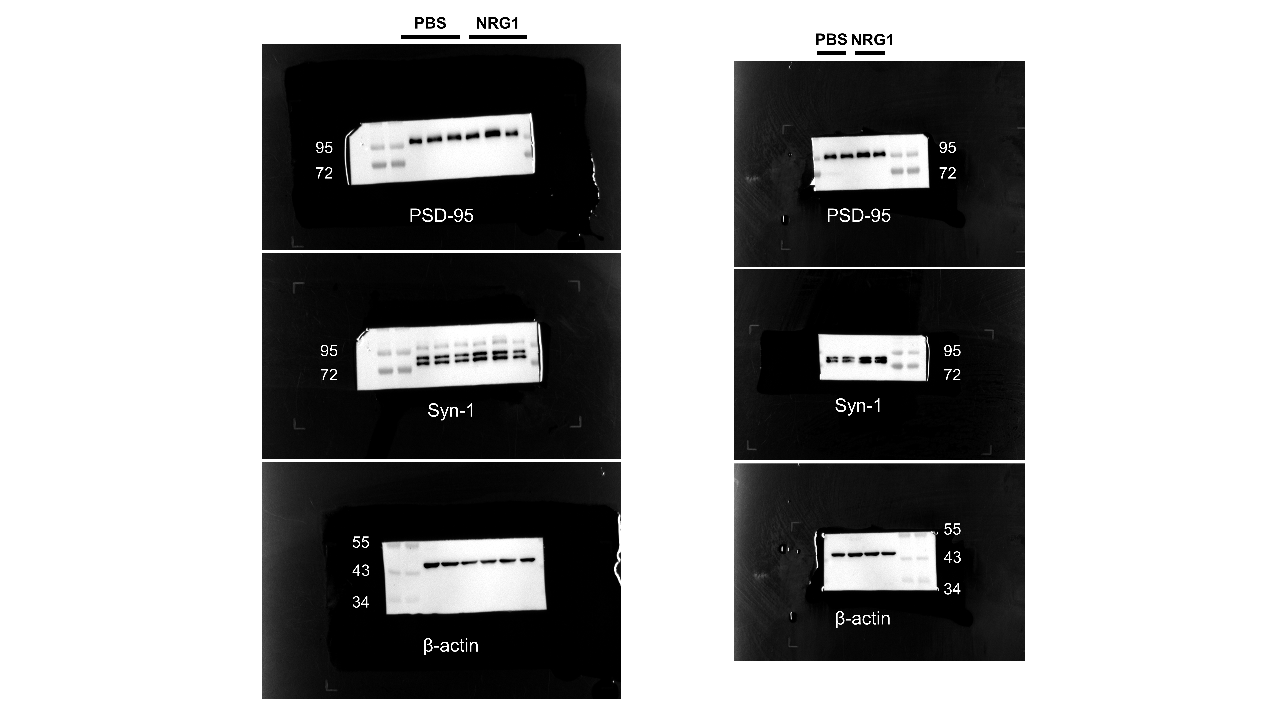


**D**

**Western blot raw images**. (A) for Fig. 4D; (B) for Fig. 5K; (C) for Fig. 5N; (D) for Fig. 6T.

Supplement: Supplementary file 2 — Supplementary Material 2. [file 12964_2024_1528_MOESM2_ESM.docx]
